# Profound and reproducible patterns of reduced regional gray matter characterize major depressive disorder

**DOI:** 10.1038/s41398-019-0512-8

**Published:** 2019-07-24

**Authors:** Sarah C. Hellewell, Thomas Welton, Jerome J. Maller, Matthew Lyon, Mayuresh S. Korgaonkar, Stephen H. Koslow, Leanne M. Williams, A. John Rush, Evian Gordon, Stuart M. Grieve

**Affiliations:** 1 Sydney Translational Imaging Laboratory, Heart Research Institute, Charles Perkins Centre, University of Sydney, Sydney, NSW 2006 Australia; 2 General Electric Healthcare, Richmond, Richmond, VIC Australia; 3 Monash Alfred Psychiatry research centre, Melbourne, VIC Australia; 4 The Brain Dynamics Centre, Westmead Institute for Medical Research and Sydney Medical School, The University of Sydney, Sydney, NSW Australia; 5 Department of Psychiatry and Behavioral Sciences, University of Miami Miller School of Medicine, Miami, FL 33136 USA; 6 Sierra-Pacific Mental Illness Research, Education, and Clinical Center (MIRECC) Veterans Affairs Palo Alto Health Care System, Palo Alto, CA 94304 USA; 7 Duke-National University of Singapore, Singapore, Singapore; 8 Department of Psychiatry, Duke Medical School, Durham, NC USA; 9 Texas Tech University-Health Sciences Center, Permian Basin, TX USA; 10 Brain Resource Ltd, Sydney, NSW, Australia and San Francisco, CA USA; 11 Department of Radiology, Royal Prince Alfred Hospital, Camperdown, Sydney, NSW 2006 Australia

**Keywords:** Depression, Diagnostic markers

## Abstract

Reduced gray matter (GM) volume may represent a hallmark of major depressive disorder (MDD) neuropathology, typified by wide-ranging distribution of structural alteration. In the study, we aimed to replicate and extend our previous finding of profound and widespread GM loss in MDD, and evaluate the diagnostic accuracy of a structural biomarker derived from GM volume in an interconnected pattern across the brain. In a sub-study of the International Study to Predict Optimized Treatment in Depression (iSPOT-D), two cohorts of clinically defined MDD participants “Test” (*n* = 98) and “Replication” (*n* = 131) were assessed alongside healthy controls (*n* = 66). Using 3T MRI T1-weighted volumes, GM volume differences were evaluated using voxel-based morphometry. Sensitivity, specificity, and area under the receiver operating characteristic curve were used to evaluate an MDD diagnostic biomarker based on a precise spatial pattern of GM loss constructed using principal component analysis. We demonstrated a highly conserved symmetric widespread pattern of reduced GM volume in MDD, replicating our previous findings. Three bilateral dominant clusters were observed: Cluster 1: midline/cingulate (GM reduction: Test: 6.4%, Replication: 5.3%), Cluster 2: medial temporal lobe (GM reduction: Test: 8.2%, Replication: 11.9%), Cluster 3: prefrontal cortex (GM reduction: Test: 12.1%, Replication: 23.2%). We developed a biomarker reflecting the global pattern of GM reduction, achieving good diagnostic classification performance (AUC: Test = 0.75, Replication = 0.84). This study establishes that a highly specific pattern of reduced GM volume is a feature of MDD, suggestive of a structural basis for this disease. We introduce and validate a novel diagnostic biomarker based on this pattern.

## Introduction

Major depressive disorder (MDD) is a significant cause of morbidity worldwide, affecting more than sixteen million Americans and three million Australians annually^1,2^. As the most frequent disease affecting young people, MDD is an immense health and economic burden, with an annual cost of $98.9 billion US^3^. Due to heterogeneity of MDD presentation, diagnosis is based on the clinical impression and application of Diagnostic and Statistical Manual of Mental Disorders (DSM) criteria^4^, with no reliable or quantifiable marker or test despite decades of active research. Using the first 50% cohort of the International Study to Predict Optimized Treatment in Depression (iSPOT-D), we previously demonstrated a widespread MDD-related gray matter (GM) volumetric change equivalent to several decades of ageing^5,6^. Here we aim to assess the replicability of these findings in the second cohort of this trial, and further evaluate whether the patterns of GM alteration might constitute a “signature” of MDD.

MDD-related GM abnormalities have been previously described using magnetic resonance imaging (MRI) and voxel-based morphometry (VBM)^7–10^. However, there is considerable contention regarding the key regions of GM structural abnormality. Volumetric reductions have largely been reported in the dorsolateral, orbitofrontal cortex (OFC) and medial prefrontal cortex^6,11–14^, anterior cingulate cortex^14,15^, insular cortex^16^, limbic system^11,17–19^, superior temporal gyrus^6^, and dorsal striatum^6,12^, however many offer conflicting findings, suggesting that MDD-related GM alterations are highly heterogeneous. Research has also been hampered by underpowered studies, which have contributed to the inconsistent findings.

Volumetric changes are particularly important in the context of MDD pathophysiology and symptomology, with many implicated regions playing central roles in high-level cognition, affective, and social behaviors via neural circuitry connecting the frontal lobe to temporal and parietal lobes^20^. MDD-related GM volumetric changes have been correlated with deficits in attentional control^21,22^, visual and spatial memory^23,24^, executive processing^25,26^, and emotional dysregulation^27,28^, suggesting that the structural consequences of MDD may be linked to psychological and functional state. While conventional MDD diagnostic methods of assessment can be routinely employed in clinical settings, such tests are largely based on clinical impression only^29^. Given the highly heterogeneous presentations and symptoms experienced in MDD, it is likely that psychological and functional tests more broadly reflect a disease syndrome, and are not indicative of the true pathological state of MDD^30^. This is particularly the case with “subclinical” MDD patients, for whom GM structural change has been observed despite negative classification using DSM criteria for MDD^31^. Despite this heterogeneity, GM alteration appears to be a constant consequence, and thus objective measurement of the underlying neurobiology is increasingly important. Given the clinical and research shift towards a precision medicine approach to diagnoses, structural biomarkers of underlying pathobiology may be useful as a complementary method of diagnosis and characterization in suspected MDD patients.

## Materials and methods

### Study design

Data were gathered from the iSPOT-D trial, described previously^32,33^. The study was approved by the Western Sydney Ethics Committee and all participants provided written informed consent. Participants were recruited from primary care, community, and academic psychiatry settings to represent a broad sample of depression treatment-seekers. The Mini-International Neuropsychiatric Interview^34^ using DSM-IV criteria^35^ and 17-item Hamilton Depression Rating Scale (HDRS_17_)^36^ score ≥ 16 confirmed the primary diagnosis of MDD.

### Participants

Data were drawn from the imaging arm of the iSPOT-D study, comprising 233 participants with confirmed diagnosis of MDD and 66 healthy controls (Supplementary Fig. 1). The MDD group was further divided into a Test MDD (*n* = 98) and Replication MDD (*n* = 131) cohort. Demographic and clinical data were obtained at baseline, including age, sex, age at first MDD diagnosis, and depression duration. A full list of inclusion and exclusion criteria are detailed in ref. ^33^. All MDD participants were either antidepressant medication-naïve or had undergone a wash-out period of at least five half-lives of a previously prescribed antidepressant. Baseline MRI sequences were obtained on all participants, as described below. A total of four T1-weighted datasets were unusable due to motion artifact.

### Imaging protocol

MRI data were acquired at baseline using a 3-Tesla GE Signa HDx scanner (GE Healthcare, Milwaukee, Wisconsin) as previously described^5^ using an 8-channel head coil. T1-weighted images were acquired using a contiguous AC-PC aligned sagittal IR-SPGR sequence (TR = 8.3 ms, TE = 3.2 ms, TI = 500 ms, flip angle = 11 degrees, matrix = 256 × 256, voxel dimensions = 1 mm isotropic, and NEX = 1).

### Whole-brain voxel-based morphometry

T1-weighted image data were preprocessed and analyzed using the CAT12 toolbox (http://dbm.neuro.uni-jena.de/cat), an extension to the SPM12 software package (http://www.fil.ion.ucl.ac.uk/spm/software/spm12). Images were normalized using affine followed by nonlinear registration, corrected for bias field inhomogeneities, then segmented into GM, white matter and cerebrospinal fluid components. Images were registered to standard MNI space using high-dimensional DARTEL normalization. Segmentation was further refined by accounting for partial volume effects, and applying a hidden Markov random field model, which incorporates spatial prior information of adjacent voxels into the segmentation estimation. The warped tissue type images were modulated to preserve the volume of a particular tissue within a voxel by multiplying voxel values in the segmented images by the Jacobian determinants derived from the spatial normalization step. Finally, images were smoothed with a full-width half-maximum kernel of 8 mm. Regions were labeled with reference to the Automated Anatomical Labeling (AAL) atlas^37^.

### Region of interest (ROI) analyses

To investigate the spatial pattern of focal GM loss, ROIs defined by 5 mm spheres were generated using MarsBaR (MRC Cognition and Brain Sciences Unit, Cambridge, United Kingdom) in SPM12. ROIs were centerd on regions identified by both t-statistic map peak voxels and visual inspection of anatomical locations with the aid of the AAL2 atlas^38^. To aid inter-subject comparison, each ROI was standardized to the mean value for that ROI, and *Z*-scores produced. To construct a diagnostic structural biomarker that faithfully represented the most pronounced anatomical structural differences in MDD, we considered the ROI-based voxel data and the correlations derived thereof to determine the regions with (a) the greatest GM differences and (b) the highest inter-regional correlations, with the rationale that a combined biomarker derived of these ROIs would represent a structurally oriented organization of diminished GM.

### Statistical analyses

Where comparison was performed between MDD and healthy control cohorts, volume and ROI differences were evaluated using two-tailed independent sample *t*-tests or one-way ANOVA. Analyses were performed at every voxel for the VBM data, with a threshold of *p* < 0.01 false discovery rate corrected for multiple comparisons. Pearson’s correlation was used to examine relationships between ROIs, the structural biomarker and clinical variables. ROC curve analyses were used to assess diagnostic capacity and discriminatory ability of the biomarker to divide MDD from control participants. The optimal cutoff point as defined by Youden’s J statistic in the Test cohort was used to examine the unrelated data of the Replication cohort, with additional ROC analysis performed on the biomarker in the Replication cohort to determine reproducibility. Statistical analyses were conducted using SPSS 25.0 (SPSS, IBM; Chicago, IL). Data are expressed as mean ± standard deviation (SD).

## Results

### Participant characteristics

Table 1 summarizes the clinical and demographic characteristics of the control (*n* = 66), Test MDD (*n* = 98), and Replication MDD (*n* = 131) cohorts. No significant difference in age, gender, or education was present between controls and either MDD cohort, while baseline HDRS_17_, disease onset and disease duration were similarly matched in both the Test and Replication cohorts, with both Test and Replication cohorts classified as moderately depressed according to the criteria outlined in ref. ^39^. Global GM volumes (corrected for total brain volume, TBV) are presented in Table 1. Both the Test (5% reduction, *p* < 0.001) and Replication (6% reduction, *p* < 0.001) cohorts had reduced global GM volume when compared with the healthy control cohort. No difference in global GM volume was detected between Test and Replication groups (*p* = 0.63).

**Table 1:** Participant demographic and clinical measures

	Control cohort		Test MDD cohort		Replication MDD cohort		Combined MDD cohort	
	*N*	%	*N*	%	*N*	%	*N*	%
Number	66	100	98	100	131	100	229	100
No. female	33	50	45	46	77	59	122	53
	Mean	SD	Mean	SD	Mean	SD	Mean	SD
Age (years)	30.9	11.7	33.3	12.6	33.2	11.0	33.9	13
Education (years)	14.7	2.6	14.3	2.8	14.2	2.7	14.2	2.7
HDRS_17_ baseline	1	1.4	21.1	3.9	21.9	3.6	21.6	3.7
Age of onset (years)	–	–	22.3	12.2	21.2	8.4	21.7	10.2
Disease duration (years)	–	–	11	10.7	12	10.2	11.5	10.4
TBV	1274	117	1214	125	1204	107	1208	115
Total GM volume	729	71	690	72	686	63	688	67
Total WM volume	545	58	523	65	517	61	520	63

Volumes reported in millimeters squared

*GM* gray matter, *HDRS*_17_ 17-item Hamilton Depression Rating Scale, *MDD* major depressive disorder, *SD* standard deviation, *TBV* total brain volume

### Replication of whole-brain VBM analysis

**Fig. 1:**
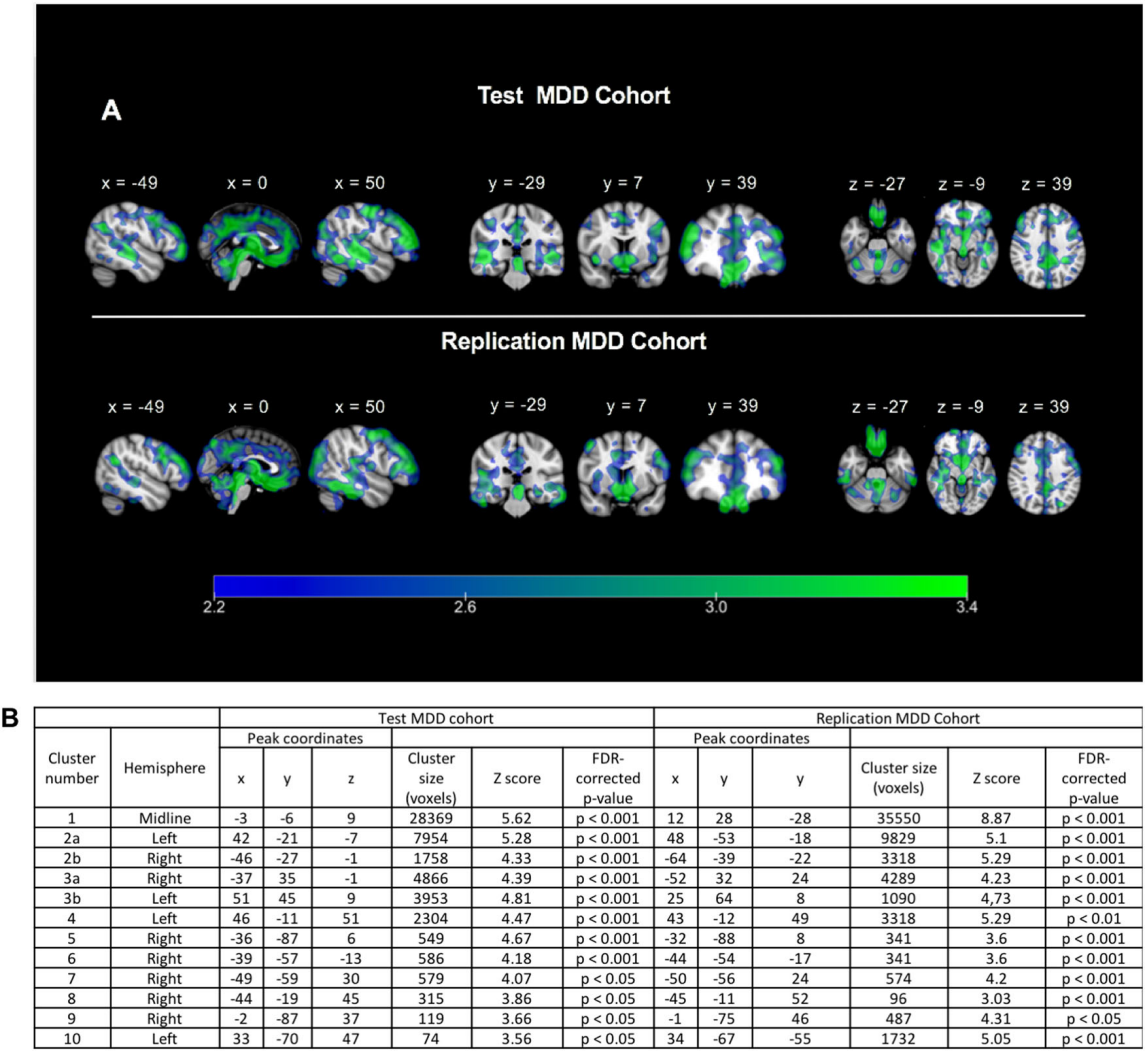
Whole-brain voxel-based morphometry revealed significant clusters of reduced GM in MDD. **a** Statistical parametric *T*-score maps of all clusters in the Test (top) and Replication (bottom) MDD cohorts, where MDD volume is significantly reduced versus control. **b** Top 10 clusters observed in the Test cohort that were successfully reproduced in the Replication MDD cohort

Figure 1 illustrates the significant clusters derived from a whole brain VBM analysis in the Test and Replication cohorts. Consistent with our prior finding^6^, we observed a widespread pattern of regionally specific reduced GM volume throughout the brain. Each of the three previously identified cluster groups were replicated in both the Test and Replication groups, with the magnitude of GM loss similar across these clusters. These three predominant clusters are described in detail below.

**Fig. 2:**
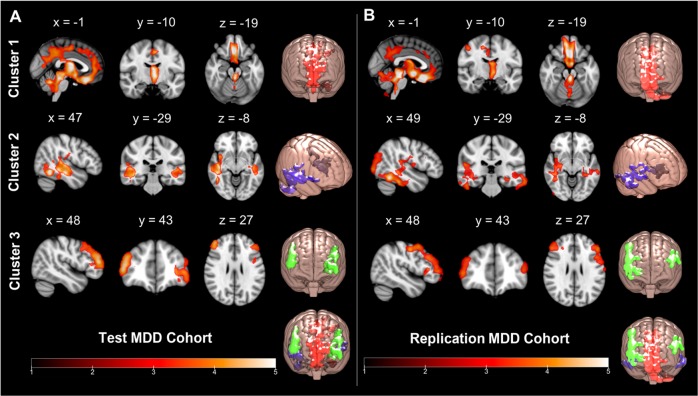
Three dominant bilateral clusters of GM alteration between MDD and control groups. Test (**a**) and Replication (**b**) cohorts. The three predominant clusters are demonstrated in columns 1–3, representing the midline structures including the cingulate bundle (Cluster 1), medial temporal lobe (Cluster 2), and prefrontal cortex (Cluster 3). The fourth column for each group shows the location of the cluster superimposed on a 3D rendering of a standard brain

The largest of these anatomical clusters (Cluster 1—“midline”) represented an average lower GM volume magnitude of 6.4% in the Test cohort and 5.3% in the Replication cohort (*p* < 0.001 for both cohorts), and was located medially, centered in the cingulate, extending posteriorly to the precuneus and cuneus, and anterioinferiorly to involve the subgenual anterior cingulate, the gyrus rectus, olfactory cortex, left thalamus, brainstem, and the cerebellum (Fig. 2, row 1). Cluster 2 (“medial temporal”) was bilateral, spanning caudally through the left and right hemispheres from the middle frontal gyrus to the insula, precentral and postcentral gyri and inferiorly to the fusiform gyrus, inferior, middle and superior temporal gyri (Fig. 2, row 2). The GM volume reduction in cluster 2 was 6.0% (L) and 2.2% (R) in the Test cohort (*p* < 0.001) and 7.1% (L) and 4.8% (R) in the Replication cohort (*p* < 0.001). Cluster 3 (“prefrontal”) was also bilateral and centered in the dorsolateral prefrontal cortex (DLPFC), involving the middle frontal gyrus and encompassing the precentral gyrus (Fig. 2, row 3). This cluster was connected medially to the midline Cluster 1. The GM volume reduction for Cluster 3 was 7.6% (L) and 4.5% (R) (*p* < 0.001, Test) and 11.5% (L) and 11.7% (*p* < 0.001, Replication) lower in GM volume compared with control.

### Structural biomarker construction in the Test MDD cohort

In order to more precisely evaluate the spatial pattern of lower GM volume, we inspected the *T*-scores in the Test cohort, identifying local maxima that corresponded to discrete anatomical structures. Supplementary Table 1 summarizes the raw GM volumes at these 43 discrete loci using 5 mm ROIs centerd at the local maximum coordinates. We then normalized raw voxel volumes for each ROI in the MDD patients to a *Z*-score, using a normal range defined by the control group. One-way ANOVA revealed significantly lower GM volume at each ROI examined in the Test MDD group. The most marked differences were found in the DLPFC (26% decrease, *p* < 0.0001), thalamus (16.3% decrease *p* < 0.0001), middle occipital gyrus (15.6% decrease, *p* < 0.0001), inferior parietal lobule (15.5% decrease, *p* < 0.0001), and cerebellar vermis (25% decrease, *p* < 0.0001). Correlation analysis was performed on normalised *Z*-scores of ROIs in the Test cohort to examine relationships between all 43 discrete GM ROIs in MDD (Supplementary Table 2, Supplementary Fig. 2A, B). ROIs were significantly positively correlated both within and between anatomically conserved regions, with the most prominent regional correlations observed between the OFC, occipital and temporal cortices, and subcortical structures. From the normalised ROI correlation analysis of *Z*-scores, we selected variables with strong positive correlation (*r* = 0.6 or greater), and produced histograms of each ROI comparing MDD and control patients to closely inspect the distribution of data. We then reproduced a correlation matrix with these ROIs (Supplementary Fig. 2C, Supplementary Table 3), and performed a principal component analysis (PCA; Supplementary Fig. 2D) to determine both the extent of variability these ROIs accounted for, and whether they classified into distinct anatomical components. PCA analysis with varimax rotation confirmed the highly conserved regional correlations between ROIs, with three distinct anatomical components accounting for 65% of the total variance: component 1 comprising 28% and components 2 and 3 each accounting for 18%.

**Fig. 3:**
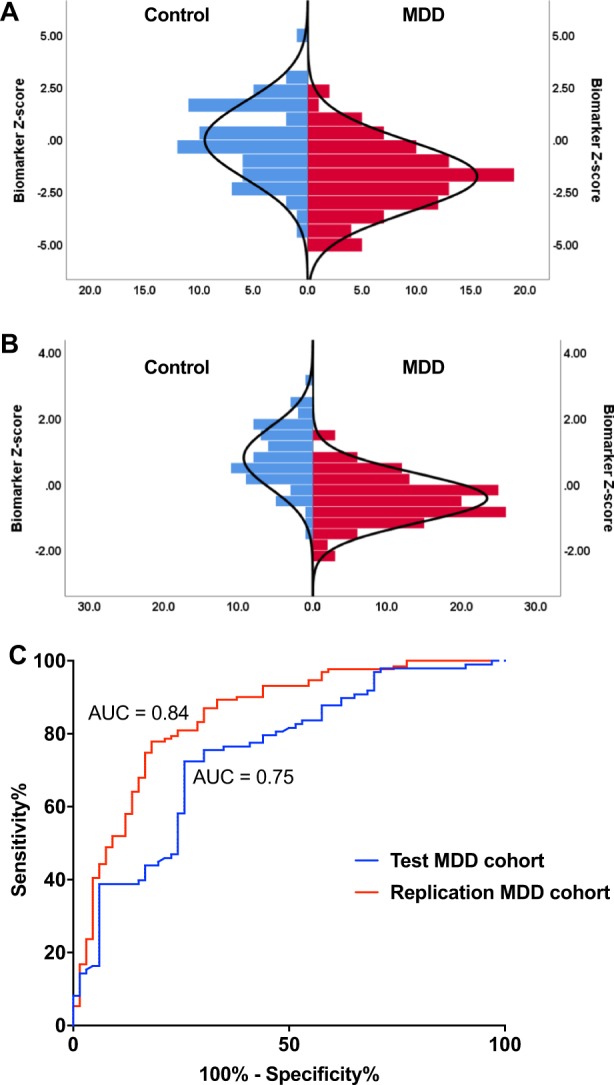
Performance of the structural biomarker in the Test and Replication MDD cohorts. **a** Normalized *Z*-scores from candidate ROIs were combined to a single structural biomarker and assessed for discriminative power in the Test MDD cohort. MDD participants had significantly lower biomarker scores compared with control participants. This finding was then reproduced in the Replication cohort (**b**), with the biomarker also demonstrating discriminative capacity in this separate patient group. Performance of the biomarker was then assessed via ROC analysis in the Test and Replication cohorts (**c**) to assess sensitivity and specificity of the biomarker

Upon confirming robust regional relationships of our prospective biomarker ROIs, we next constructed a single biomarker by combining each ROI into a single variable and converting the combined normalised ROI score to *Z*-score, and verifying statistical significance between the Test MDD and control groups (*p* < 0.0001, Fig. 3a). In the Test cohort, males exhibited lower biomarker *Z*-scores (*p* < 0.001). The biomarker was negatively correlated with age at MRI (*r*[96] = −0.40, *p* < 0.0001) and age of MDD onset (*r*[96] = −0.34, *p* < 0.01). To explore this, MDD participants were divided further into childhood or adolescent onset (≤17), young adult^16–24^, adult onset^5,24–38^, or older adult onset (>40)^40^. Age of onset over 40 was negatively correlated with biomarker score (*r*[98] = −0.30, *p* < 0.002), while no significant difference was found for any other age group. MDD participants also were grouped similarly according to age of visit (<25, 25–40, and >40), with a positive correlation only for those aged under 25 (*r*[98] = 0.47, <0.03). Next, diagnostic utility of the biomarker was assessed via ROC curve analysis, for which the area under the curve (AUC) was 0.75 (Fig. 3c). Optimal discrimination value was determined by Youden’s J statistic as a *Z*-score of 0.17, at which point sensitivity was 0.71 and specificity was 0.80.

### Structural biomarker validation in the Replication MDD cohort

To assess the diagnostic capacity of the structural biomarker identified in the Test cohort, results were validated in a separate, larger group of MDD participants in the Replication cohort, with the same anatomical ROIs contributing to the biomarker. As for the Test cohort, the biomarker was statistically significant between the Replication and Control cohorts (*p* < 0.0001, Fig. 3b). Using the predefined discrimination value of 0.17 as identified via Test cohort, 115 participants were classified as MDD-positive, while 82 were classed as MDD-negative. In the biomarker-positive group, 78% of participants were true MDD participants, while in the negatively identified group, 80% were correctly identified as controls, outperforming sensitivity and confirming specificity as defined in the Test cohort. Separate ROC analysis on the Replication cohort had an AUC of 0.84 (Fig. 3c), with sensitivity of 0.77 and specificity of 0.79. As for the Test cohort, the biomarker was negatively correlated with both age at MRI (*r*[129] = 0.46, *p* < 0.0001) and age of MDD onset (*r*[129] = −0.32, *p* < 0.001). The biomarker was also significantly related to gender, with lower mean scores for males (*p* < 0.001).

## Discussion

In this paper, we present a successful replication of our previous findings showing that a profound interconnected anatomical pattern of reduced GM volume is associated with a diagnosis of MDD^6^. Our data firmly establish that the pathophysiology of MDD has an important structural component, underpinned by several highly correlated regions of GM susceptibility. We suggest that this structural finding may be reflective of a primary anatomical MDD-related vulnerability, and/or secondary pathological change. These findings are important as they imply that a structural biomarker of GM alteration may have potential diagnostic utility, providing crucial information as to the underlying pathological state.

The etiology of MDD has been attributed to myriad causes, such as prolonged stress and hypothalamic–pituitary–adrenal axis dysfunction, neurotransmitter insufficiency, reductions in growth factors (such as brain-derived neurotrophic factor or nerve growth factor), or other hereditable factors^30,41^. The fact that such vast numbers of causalities have been identified speaks to the complexity and heterogeneity of MDD, with no singular explanation^42^. In light of this, it is increasingly important to elucidate the underlying pathophysiological changes in well-characterized populations in order to describe etiologically-specific pathology. Volumetric-based MRI analysis is a frequently employed tool to investigate GM volume change, however findings have often been inconsistent with regards to regions of change and degree of loss in MDD^17^. These factors may be partially explained by differing patient demographics and MDD clinical history and imaging protocols, but are also largely due to modest patient populations, which may be insufficiently powered to detect subtle volumetric differences^43^. In order to define definitive changes, high-powered studies are necessary.

While both our Test and Replication cohorts presented with widespread GM structural alterations, several key regions represented a primary organization of structural anomalies in MDD. These regions included the OFC, anterior cingulate, gyrus rectus, dorsal striatum (caudate, putamen), thalamus and temporal and occipital gyri, all of which have been previously implicated as regions of volumetric change in MDD^6^. Seminal work by Drevets et al. highlighted a key role of the OFC in MDD (reviewed in ref. ^44^), with reductions in GM volume coinciding with alterations in hemodynamics in the context of emotional processing, memory, and reward^45–47^. GM volume reductions in the OFC have also recently been implicated in depression-related insomnia^48^, while GM alterations of the superior and middle frontal gyri, anterior cingulate, and OFC have also been implicated in MDD-related poor sleep quality. Shifts in OFC nonreward circuitry have recently been demonstrated in resting-state functional connectivity studies^49^, with enhanced functional connectivity between the OFC, precuneus, DLPFC, and temporal gyri^50^ (which are among other ROIs we found to be different in GM volumes) suggested as a potential driver of negative sense of self and low self-esteem in MDD. While we did not examine network-based alterations and their potential effects on regional volumetric changes, prominent structural relationships have been demonstrated between the OFC, anterior cingulate, and gyrus rectus in the context of MDD-related GM alteration^31,51^, with these regions also exhibiting substantial MDD-related changes in glucose metabolism^52^. In acute-onset severe MDD, probabilistic tractography has also demonstrated altered connectivity in regions of the GM alteration in the dorsal striatum and thalamus to frontal and temporal lobes^53^. Taken together, these findings provide preliminary evidence that widespread GM volumetric change may either predicate or represent a downstream secondary consequence of the cognitive, somatic psychological manifestations of MDD. Thus, the power of our structural biomarker lies in objectively discriminating this pathological state in MDD patients irrespective of standard diagnostic criteria.

There are several limitations which may hamper interpretation of our findings. First, while results from our earlier study were reproduced in a separate patient cohort, this was conducted in the same laboratory and our findings ought to be validated by external research groups. Second, our Test and Replication cohorts were assessed in comparison with a common control group. Although unlikely, results may have been influenced by a characteristic of this control group. Third, we cannot be certain whether the observed effects are due to a true atrophic pathological state. Fourth, we have not considered the impact of previous medication regimes and how those might affect brain structure.

In this study, we describe a structural biomarker of GM alteration which reflects a highly conserved anatomical pattern of volumetric GM change, and represents a promising diagnostic biomarker of MDD. We also successfully replicated our prior findings of widespread GM alteration and demonstrated the same pattern of structural change in a separate, larger MDD cohort.

## Supplementary information

CONSORT flow chart of iSPOT-D trial

Peak region of Interest (ROI) coordinates and GM volume in the Test and Replication MDD cohorts where *p* < 0.05

Correlation of all 43 ROIs examined in the Test MDD cohort

Correlations of candidate ROIs comprising the structural biomarker

Construction of structural biomarker in the test cohort
