# Supplementary figures and images for: Profound and reproducible patterns of reduced regional gray matter characterize major depressive disorder
Source: Transl Psychiatry. 2019 Jul 24;9:176. doi: 10.1038/s41398-019-0512-8 (PMC6656728; doi:10.1038/s41398-019-0512-8)

**
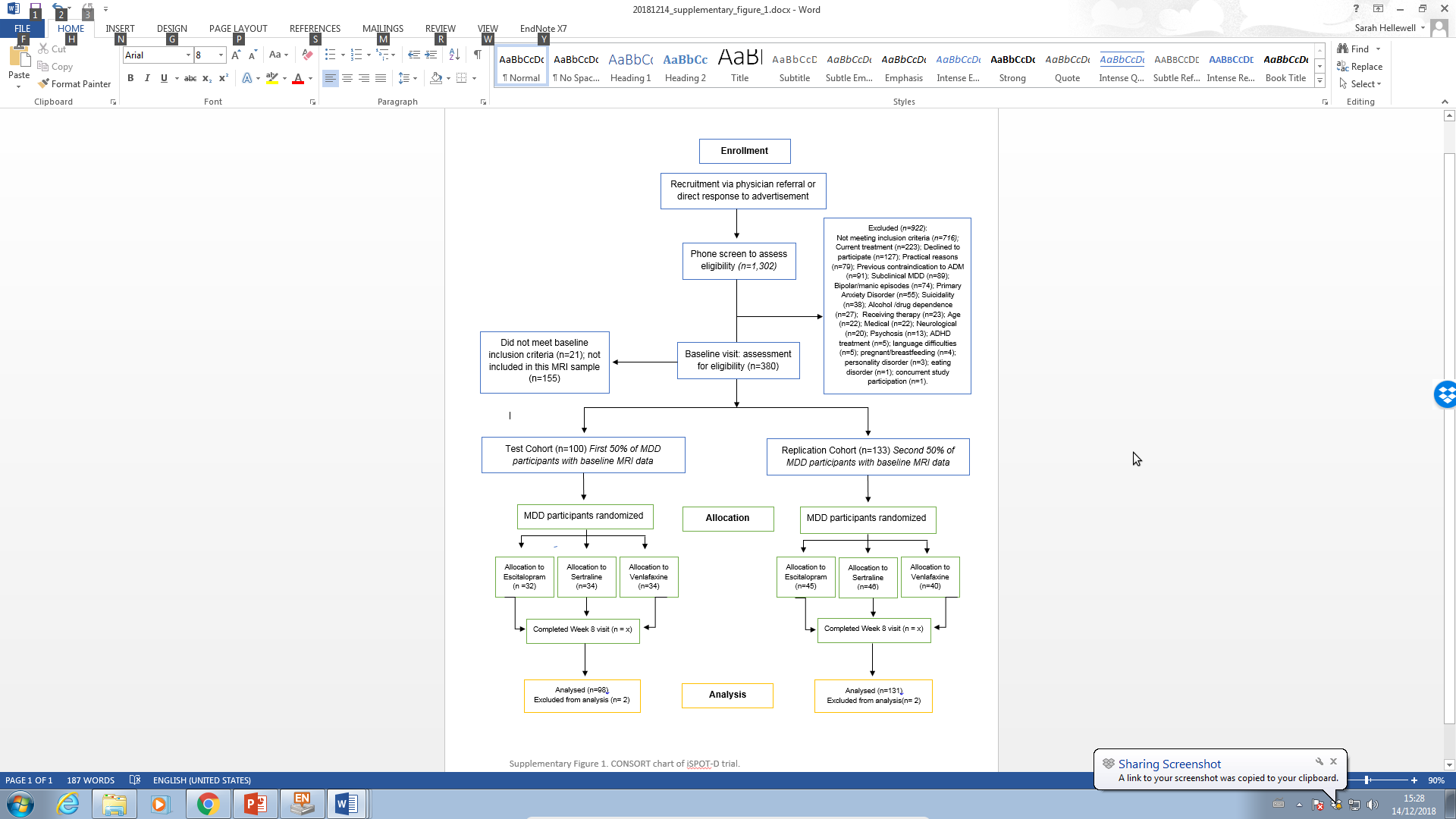
**

**Supplementary Figure 1.** CONSORT flow chart of iSPOT-D trial.

Supplement: Supplementary file 1 — CONSORT flow chart of iSPOT-D trial [file 41398_2019_512_MOESM1_ESM.docx]
