# Supplementary material for: Profound and reproducible patterns of reduced regional gray matter characterize major depressive disorder
Source: Transl Psychiatry. 2019 Jul 24;9:176. doi: 10.1038/s41398-019-0512-8 (PMC6656728; doi:10.1038/s41398-019-0512-8)
Supplement: Supplementary file 2 — Peak region of Interest (ROI) coordinates and GM volume in the Test and Replication MDD cohorts where p < 0.05 [file 41398_2019_512_MOESM2_ESM.docx]

**Supplementary Table 1.** Peak region of Interest (ROI) coordinates and GM volume in the Test and Replication MDD cohorts where p < 0.05.

| **ROI** | | **MNI coordinates** | | | **Voxels – Test cohort** | **t value** | **p value** | **% change** | **Voxels – Replication cohort** | **t value** | **p value** | **% change** |
| --- | --- | --- | --- | --- | --- | --- | --- | --- | --- | --- | --- | --- |
|  |  | **x** | **y** | **z** |  |  |  |  |  |  |  |  |
| Inferior Frontal Gyrus Triangular (L) | Control | -38 | 34 | 2 | 33.37 | 2.71 | *0.008* | 10.74 | 58.77 | 5.39 | *< 0.0001* | 15.8 |
|  | MDD |  |  |  | 29.79 |  |  |  | 49.48 |  |  |  |
| Inferior Frontal Gyrus Triangular (R) | Control | 54 | 44 | 4 | 45.53 | 4.36 | *<0.0001* | 12.82 | 61.33 | 4.91 | *< 0.0001* | 13.4 |
|  | MDD |  |  |  | 39.69 |  |  |  | 53.11 |  |  |  |
| Middle Frontal Gyrus (L) | Control | -36 | 46 | -10 | 74.88 | 3.87 | *<0.0001* | 10.29 | 89.35 | 2.84 | *< 0.05* | 4.16 |
|  | MDD |  |  |  | 67.18 |  |  |  | 85.63 |  |  |  |
| Middle Frontal Mid Orbital (L) | Control | -20 | 50 | -21 | 63.78 | 2.78 | *0.006* | 7.08 | 50.44 | 5.07 | *< 0.0001* | 13.88 |
|  | MDD |  |  |  | 59.27 |  |  |  | 43.44 |  |  |  |
| Medial Orbital Superior Frontal Gyrus (L) | Control | -3 | 46 | 9 | 114.01 | 3.74 | *<0.0001* | 9.57 | 114.01 | 4.56 | *< 0.0001* | 10.38 |
|  | MDD |  |  |  | 103.10 |  |  |  | 102.17 |  |  |  |
| Medial Orbital Superior Frontal Gyrus (R) | Control | 1 | 42 | -9 | 111.41 | 3.87 | *<0.0001* | 9.37 | 100.97 | 5.07 | *< 0.0001* | 19.69 |
|  | MDD |  |  |  | 100.97 |  |  |  | 81.10 |  |  |  |
| Superior Frontal Gyrus (R) | Control | -14 | 56 | 5 | 22.24 | 4.12 | *<0.0001* | 13.98 | 22.24 | 2.46 | *0.016* | 17.27 |
|  | MDD |  |  |  | 19.13 |  |  |  | 18.40 |  |  |  |
| Medial Superior Frontal Gyrus (L) | Control | -16 | 42 | 26 | 22.00 | 4.62 | *<0.0001* | 26.04 | 22.24 | 2.44 | *0.012* | 13.26 |
|  | MDD |  |  |  | 16.27 |  |  |  | 19.29 |  |  |  |
| Medial Superior Frontal Gyrus (R) | Control | 15 | 70 | -6 | 71.89 | 3.4 | *0.001* | 7.77 | 58.79 | 5.21 | *< 0.0001* | 12.71 |
|  | MDD |  |  |  | 66.30 |  |  |  | 51.32 |  |  |  |
| Gyrus Rectus (L) | Control | 3 | -47 | 33 | 105.70 | 5.41 | *<0.0001* | 13.38 | 49.89 | 6.75 | *< 0.0001* | 16.68 |
|  | MDD |  |  |  | 91.56 |  |  |  | 41.57 |  |  |  |
| Gyrus Rectus (R) | Control | 6 | 31 | -17 | 75.78 | 4.26 | *<0.0001* | 10.45 | 106.60 | 7.73 | *< 0.0001* | 17.3 |
|  | MDD |  |  |  | 67.86 |  |  |  | 88.16 |  |  |  |
| Anterior Cingulate (L) | Control | -2 | 47 | -4 | 98.25 | 3.73 | *<0.0001* | 9.91 | 61.02 | 4.32 | *< 0.0001* | 16.06 |
|  | MDD |  |  |  | 88.52 |  |  |  | 51.22 |  |  |  |
| Medial Cingulate (R) | Control | 3 | -47 | 33 | 113.77 | 3.81 | *<0.0001* | 9.74 | 65.49 | 3.96 | *< 0.0001* | 7.92 |
|  | MDD |  |  |  | 102.69 |  |  |  | 60.30 |  |  |  |
| Precuneus (L) | Control | -2 | -48 | 45 | 88.40 | 3.99 | *<0.0001* | 10.10 | 33.99 | 3.98 | *< 0.0001* | 13.65 |
|  | MDD |  |  |  | 79.47 |  |  |  | 29.35 |  |  |  |
| Supplementary Motor Area (L) | Control | -10 | 12 | 57 | 39.44 | 2.8 | *0.005* | 8.70 | 72.55 | 2.90 | *0.04* | 3.75 |
|  | MDD |  |  |  | 36.01 |  |  |  | 69.83 |  |  |  |
| Precentral Gyrus (L) | Control | -45 | -2 | 40 | 74.63 | 3.39 | *0.001* | 12.66 | 63.07 | 5.38 | *< 0.0001* | 13.37 |
|  | MDD |  |  |  | 65.18 |  |  |  | 54.64 |  |  |  |
| Precentral Gyrus (R) | Control | 46 | -10 | 51 | 70.00 | 4.67 | *<0.0001* | 14.78 | 71.11 | 4.62 | *< 0.0001* | 12.84 |
|  | MDD |  |  |  | 59.66 |  |  |  | 61.98 |  |  |  |
| Fusiform Gyrus (L) | Control | -39 | -58 | -12 | 84.09 | 3.87 | *<0.0001* | 12.18 | 119.63 | 5.06 | *< 0.0001* | 9.72 |
|  | MDD |  |  |  | 73.85 |  |  |  | 108.00 |  |  |  |
| Inferior Temporal Gyrus (L) | Control | -44 | -54 | -16 | 124.89 | 3.95 | *<0.0001* | 12.22 | 70.68 | 5.01 | *<0.0001* | 16.54 |
|  | MDD |  |  |  | 109.64 |  |  |  | 58.99 |  |  |  |
| Inferior Temporal Gyrus (R) | Control | 50 | -51 | -14 | 89.57 | 5.26 | *<0.0001* | 13.83 | 108.90 | 5.58 | *<0.0001* | 13.01 |
|  | MDD |  |  |  | 77.19 |  |  |  | 94.73 |  |  |  |
| Middle Temporal Gyrus (L) | Control | -46 | -27 | 0 | 56.63 | 3.73 | *<0.0001* | 8.17 | 93.78 | 4.45 | *<0.0001* | 13.18 |
|  | MDD |  |  |  | 52.00 |  |  |  | 81.42 |  |  |  |
| Middle Temporal Gyrus (R) | Control | 51 | -58 | 0 | 87.34 | 3.95 | *<0.0001* | 13.11 | 87.33 | 1.87 | *0.04* | 6.14 |
|  | MDD |  |  |  | 75.89 |  |  |  | 81.97 |  |  |  |
| Superior Temporal Gyrus (R) | Control | 43 | -10 | -9 | 77.10 | 3.55 | *0.001* | 7.52 | 67.02 | 4.44 | *<0.0001* | 11.16 |
|  | MDD |  |  |  | 71.30 |  |  |  | 59.54 |  |  |  |
| Postcentral Gyrus (L) | Control | -44 | -20 | 45 | 52.44 | 3.23 | *0.002* | 9.64 | 53.64 | 4.43 | *<0.0001* | 16.52 |
|  | MDD |  |  |  | 47.38 |  |  |  | 44.78 |  |  |  |
| Postcentral Gyrus (R) | Control | 42 | -34 | 50 | 74.32 | 3.66 | *<0.0001* | 10.82 | 74.31 | 2.17 | *0.03* | 6.84 |
|  | MDD |  |  |  | 66.27 |  |  |  | 69.23 |  |  |  |
| Inferior Parietal (L) | Control | -30 | -40 | 40 | 69.02 | 4.20 | *<0.0001* | 15.46 | 69.02 | 3.0 | *0.003* | 11.05 |
|  | MDD |  |  |  | 58.35 |  |  |  | 61.39 |  |  |  |
| Angular Gyrus (L) | Control | -48 | -62 | 26 | 85.59 | 3.86 | *<0.0001* | 11.68 | 102.25 | 4.48 | *<0.0001* | 13.13 |
|  | MDD |  |  |  | 75.59 |  |  |  | 88.82 |  |  |  |
| Angular Gyrus (R) | Control | 50 | -75 | 32 | 47.41 | 4.36 | *<0.0001* | 11.09 | 64.32 | 4.61 | *<0.0001* | 10.63 |
|  | MDD |  |  |  | 42.15 |  |  |  | 57.48 |  |  |  |
| Cuneus (L) | Control | -2 | -88 | 38 | 49.42 | 4.38 | *<0.0001* | 12.61 | 60.93 | 4.8 | *<0.0001* | 12.85 |
|  | MDD |  |  |  | 43.19 |  |  |  | 53.10 |  |  |  |
| Cuneus (R) | Control | 6 | -93 | 14 | 73.71 | 3.88 | *<0.0001* | 13.63 | 67.57 | 2.97 | *0.003* | 10.60 |
|  | MDD |  |  |  | 63.66 |  |  |  | 60.41 |  |  |  |
| Lingual Gyrus (L) | Control | -14 | -90 | -3 | 57.81 | 3.86 | *<0.0001* | 12.09 | 52.17 | 1.90 | *0.04* | 5.65 |
|  | MDD |  |  |  | 50.82 |  |  |  | 49.22 |  |  |  |
| Calcarine F (R) | Control | 22 | -80 | 14 | 32.93 | 3.38 | *0.001* | 18.99 | 32.92 | 1.89 | *0.04* | 8.78 |
|  | MDD |  |  |  | 26.67 |  |  |  | 30.03 |  |  |  |
| Inferior Occipital Gyrus (R) | Control | 45 | -87 | -3 | 70.53 | 4.01 | *<0.0001* | 11.99 | 67.89 | 5.15 | *<0.0001* | 13.1 |
|  | MDD |  |  |  | 62.07 |  |  |  | 59.00 |  |  |  |
| Middle Occipital Gyrus (L) | Control | -34 | -87 | 6 | 112.98 | 4.97 | *<0.0001* | 15.60 | 69.45 | 4.78 | *<0.0001* | 12.63 |
|  | MDD |  |  |  | 95.36 |  |  |  | 60.68 |  |  |  |
| Hippocampus (L) | Control | -36 | -34 | -10 | 53.66 | 2.53 | *0.01* | 6.20 | 73.13 | 4.30 | *<0.0001* | 8.7 |
|  | MDD |  |  |  | 50.33 |  |  |  | 66.77 |  |  |  |
| Thalamus (L) | Control | -3 | -7 | 4 | 98.08 | 5.48 | *<0.0001* | 16.27 | 98.02 | 4.65 | *<0.0001* | 12.53 |
|  | MDD |  |  |  | 82.13 |  |  |  | 85.74 |  |  |  |
| Caudate (L) | Control | -3 | 6 | 5 | 106.83 | 4.01 | *<0.0001* | 9.84 | 117.13 | 6.72 | *<0.0001* | 12.34 |
|  | MDD |  |  |  | 96.31 |  |  |  | 102.68 |  |  |  |
| Caudate (R) | Control | 10 | 7 | -9 | 108.79 | 4.03 | *<0.0001* | 6.85 | 112.88 | 3.94 | *<0.0001* | 10.1 |
|  | MDD |  |  |  | 101.34 |  |  |  | 101.48 |  |  |  |
| Putamen (L) | Control | -10 | 12 | 57 | 115.44 | 4.02 | *<0.0001* | 8.20 | 112.88 | 5.58 | *< 0.0001* | 10.1 |
|  | MDD |  |  |  | 105.97 |  |  |  | 101.48 |  |  |  |
| Insula (L) | Control | -31 | 26 | 1 | 112.27 | 3.64 | *<0.0001* | 8.39 | 112.27 | 2.54 | *0.01* | 9.61 |
|  | MDD |  |  |  | 102.86 |  |  |  | 101.48 |  |  |  |
| Insula (R) | Control | 34 | 18 | -12 | 125.83 | 3.80 | *<0.0001* | 7.40 | 58.22 | 4.63 | *<0.0001* | 10.05 |
|  | MDD |  |  |  | 116.52 |  |  |  | 52.37 |  |  |  |
| Vermis 1 2 (R) | Control | 1 | -37 | -22 | 30.44 | 4.40 | *<0.0001* | 25.06 | 57.73 | 3.73 | *< 0.001* | 7.57 |
|  | MDD |  |  |  | 22.81 |  |  |  | 53.36 |  |  |  |
| Cerebellum Crus 1 (L) | Control | -30 | -64 | -33 | 119.15 | 3.63 | *<0.0001* | 7.86 | 125.96 | 4.21 | *<0.0001* | 7.9 |
|  | MDD |  |  |  | 109.78 |  |  |  | 116.01 |  |  |  |
| Cerebellum Lobule 9 (L) | Control | -10 | 12 | 57 | 51.64 | 3.41 | *0.001* | 9.72 | 51.64 | 2.49 | *0.01* | 6.91 |
|  | MDD |  |  |  | 46.62 |  |  |  | 48.07 |  |  |  |
