# Supplementary material for: Profound and reproducible patterns of reduced regional gray matter characterize major depressive disorder
Source: Transl Psychiatry. 2019 Jul 24;9:176. doi: 10.1038/s41398-019-0512-8 (PMC6656728; doi:10.1038/s41398-019-0512-8)
Supplement: Supplementary file 3 — Correlation of all 43 ROIs examined in the Test MDD cohort [file 41398_2019_512_MOESM3_ESM.docx]

**Supplementary Table 2.** Correlation of all 43 ROIs examined in the Test MDD cohort.
