# Supplementary material for: Profound and reproducible patterns of reduced regional gray matter characterize major depressive disorder
Source: Transl Psychiatry. 2019 Jul 24;9:176. doi: 10.1038/s41398-019-0512-8 (PMC6656728; doi:10.1038/s41398-019-0512-8)
Supplement: Supplementary file 5 — Construction of structural biomarker in the test cohort [file 41398_2019_512_MOESM5_ESM.docx]

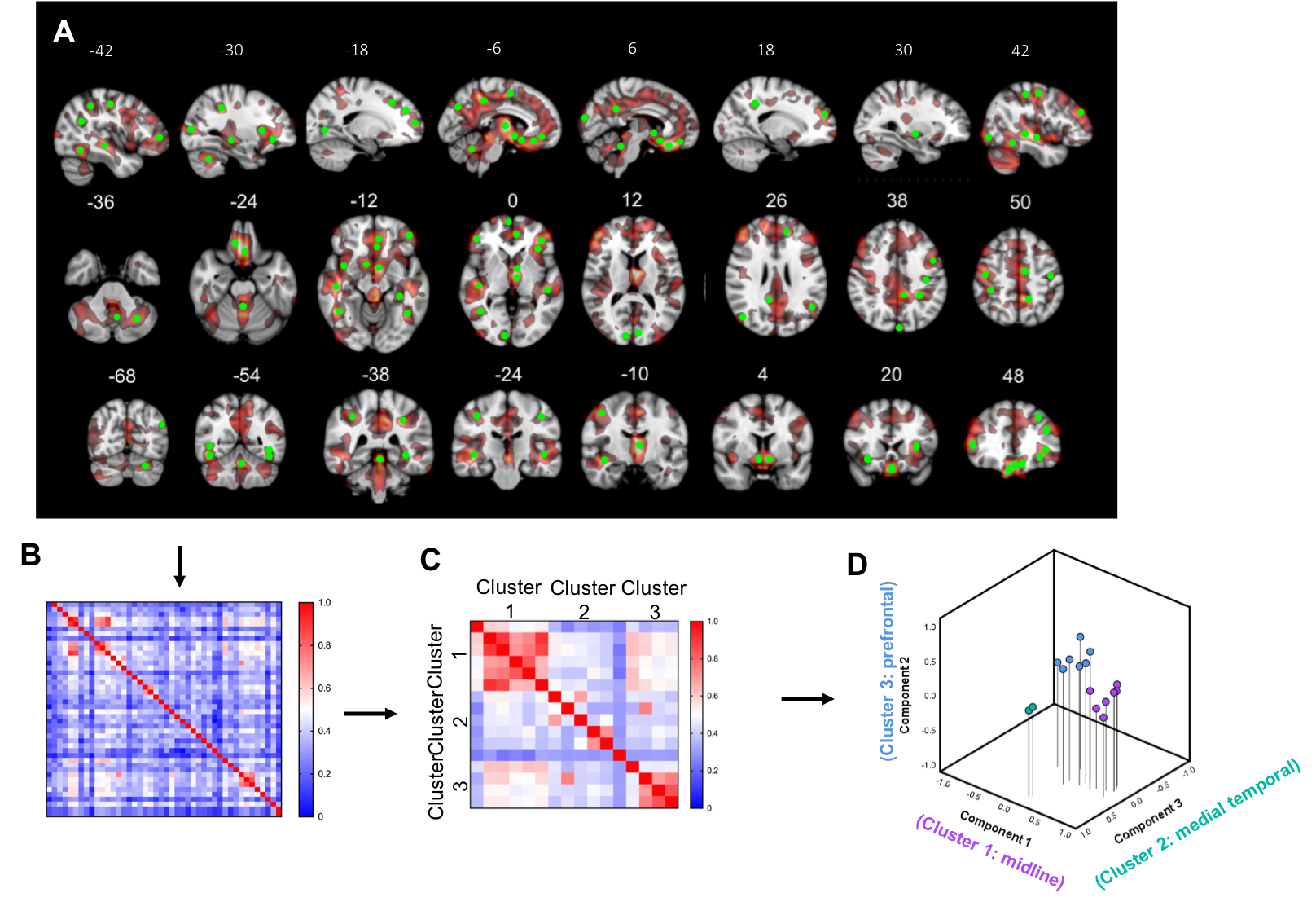


**Supplementary Figure 2. Construction of structural biomarker in the test cohort**. (A) spherical ROIs centered on local cluster maxima in Test and Replication MDD cohorts vs. healthy control participants. (B) Heat map displaying Pearson correlation (r) coefficients in the Test cohort between all 43 ROIs with strength and directionality of relationships ranging from 0 to +1 coded in blue to pink, respectively. (C) Heat map of candidate biomarker ROIs selected on the basis of high correlation within anatomical regions (D) PCA of ROIs confirmed 3 anatomical components corresponding to clusters 1, 2 & 3. The Z scores of these ROIs were combined as a single variable for use as a structural biomarker.
